# Editorial: Rising stars in clinical microbiology: 2022

**DOI:** 10.3389/fcimb.2022.1066181

**Published:** 2022-11-14

**Authors:** Rafael Franco

**Affiliations:** ^1^ CiberNed Network Center for Neurodegenerative Diseases, National Spanish Health Institute Carlos III, Madrid, Spain; ^2^ Molecular Neurobiology Laboratory, Department of Biochemistry and Molecular Biomedicine, Faculty of Biology, Universitat de Barcelona, Barcelona, Spain; ^3^ School of Chemistry, Universitat de Barcelona, Barcelona, Spain

**Keywords:** SARS-CoV-2, microbiota, viral infection, cancer, constipation, rheumatoid arthritis, probiotics

The objective of this Research Topic was to highlight “rising stars” in what concerns new approaches in microbiology research that address pending issues and, more importantly, that arise in response to new challenges such as the one posed by the SARS-CoV-2 pandemic. In addition, one of the articles, through a meta-analysis, underscores the possible relationship between the gram negative bacterium *Porphyromonas gingivalis* and a common age-related disease involving alterations in the immunological response, namely rheumatoid arthritis (Li et al.). Altered immunological responses caused by age, drug use, environmental factors, etc. may be incited and/or aggravated by microorganisms. One of the most popular articles, with >1800 views as of today (October 10, 2022), is devoted to the potential of vitamin D to combat inflammation in macrophages in patients with Crohn’s disease who are also infected with *Mycobacterium avium subsp. paratuberculosis* (Vaccaro et al). Another article, a review with >2,200 views, deals with the anti-cancer potential of oncolytic viruses. This review suggests that gastrointestinal tumors can be managed more effectively if immunotherapy takes into account the synergistic action of oncolytic viruses with immunoregulatory power (Li et al.).

In further confirmation of the correlation between the intestinal microbiota and disease risk and/or its impact on disease progression, the Research Topic contains multiple highly compelling studies and reviews on this subject. One of the reviews highlights the possibility that probiotics may decrease the risk of viral infection. Multiple mechanisms may be in operation, from the probiotic triggering of anti-viral immune cells to the production of antiviral metabolites and binding of these compounds to viruses to prevent cell infection. The article includes a table presenting the effects of common bacteria present in probiotic preparations in terms of preparing the human body to fight invading viral pathogens (Wang et al.). *Akkermansia muciniphila*, a commensal bacterium present in the intestine, is beneficial in the maintenance of a healthy microflora and a balanced gut immune system (see Rodrigues et al., 2022, for a review). Interestingly, Lv et al. decipher the genomic architecture of *Akkermansia* species found in the human intestine, also identifying different phylogroups when comparing data obtained from Chinese and Western populations. The authors also demonstrate the presence of a strain, *A. glicaniphila GP37*, previously described only in python (Lv et al.). The relevance of a proper microbiota is highlighted in another paper, in which alteration of bile acid metabolism is observed in patients with slow transit constipation. The authors conclude that their study “*provides an in-depth understanding of the relationship between the fecal microbiota, metabolites, and intestinal dysfunction in slow transit constipation patients*” (Fan et al.).

Microbiology is a common source of novel techniques to detect infections. This calls to mind the difficulties of obtaining the virus causing hepatitis D *via* PCR. The causal factor for hepatitis D is a viroid with a high complementarity of RNA sequences, resulting in a compact structure with a high degree of complementarity: that is, with bases that form a tight double helix. This structure is so difficult to relax to allow the RNA polymerase to act in a PCR assay that it is necessary to boil the sample for 15 min to achieve success (F. Rodriguez-Frías, personal communication; see details in Schaper et al., 2010 and Homs et al., 2014). Although there are RNAs that are very fragile, that of the viroid that causes hepatitis D in fact remains undegraded under adverse conditions. In the single methodological article included in the Research Topic, a luciferase-based methodology is used to detect antibodies against swine fever virus. The method consists of immunoprecipitation and, to increase detection sensitivity, takes advantage of the high yield of Gaussia luciferase, which is obtained from *Gaussia princeps*, a marine copepod (Ding et al.,).

The Research Topic also includes an original research article and a review related to SARS-CoV-2 coronavirus infection. The review summarizes the primary mechanisms involved in severe cases of respiratory distress in patients with COVID-19. Among the novel contributions of this article is a description of the involvement of vitamin D in its role as an immunomodulator. It seems that patients who have low levels of blood vitamin D have poor prognosis; however, it is not demonstrated whether vitamin supplementation helps patients with COVID-19 (Zheng et al.). The original research article reports on the isolation and testing of chimeric virus-like particles that contain proteins coded by the SARS-CoV-2 genome. The authors prepared a plasmid that contained the sequence of the spike protein, which interacts with the virus receptor at host cells, in two forms: one intact, and another as a spike–H5N1 protein fusion, H5N1 being a protein of the avian influenza A virus. Virus-like particles containing these proteins, produced in a “*baculovirus insect cell expression system*,” were found to be immunogenic in mice. These results indicate that the approach may be useful in the design of novel anti-SARS-CoV-2 vaccines (Chen et al.).

Finally, the Research Topic does not lack discussion of novel and sophisticated techniques: Dai et al. propose the popular CRISPR/Cas9 system as a method of preparing a defective form of BHV-1 bovine herpesvirus virus, which causes vulvovaginitis and rhinotracheitis in cattle. The defective virus could eventually be used to prepare a vaccine, but at present has served to demonstrate that the UL41 viral gene is important for replication. Bioinformatics approaches show value in the construction of a model of the *Fiber* protein (gene ON164651) of the CAdV-1 canine adenovirus. The model has served to predict KLGVKPTTY as the amino acid sequence that binds to the major histocompatibility complex-1 in lymphocytes, thus paving the way for the development of an epitope-based vaccine (Wang et al.).

## Author contributions

The author confirms being the sole contributor of this work and has approved it for publication.

## Conflict of interest

The author declares that the research was conducted in the absence of any commercial or financial relationships that could be construed as a potential conflict of interest.

## Publisher’s note

All claims expressed in this article are solely those of the authors and do not necessarily represent those of their affiliated organizations, or those of the publisher, the editors and the reviewers. Any product that may be evaluated in this article, or claim that may be made by its manufacturer, is not guaranteed or endorsed by the publisher.
